# Bacteriological profile of community peritonitis operated in a Moroccan Hospital

**DOI:** 10.1099/acmi.0.000816.v5

**Published:** 2024-10-24

**Authors:** Samia Bazhar, Elmostafa Benaissa, Fatima Ziad, Leila Laamara, Yassine Ben Lahlou, Mariama Chadli, Mostafa Elouennass

**Affiliations:** 1 Department of Clinical Bacteriology, Mohammed V Military Teaching Hospital, Faculty of Medicine and Pharmacy, Mohammed V University in Rabat, Rabat, Morocco; 2 Research Team of Epidemiology and Bacterial Resistance, Faculty of Medicine and Pharmacy, Mohammed V University in Rabat, Rabat, Morocco

**Keywords:** antibiotic, community, peritonitis, resistance

## Abstract

**Introduction.** Peritonitis is characterized by acute inflammation of the peritoneum, often resulting from digestive organ perforation or intra-abdominal septic focus. It may be of either infectious or non-infectious origin. The bacteria involved are those of the digestive flora (*Enterobacteriaceae* and anaerobes), while Gram-positive cocci and yeasts can be isolated in nosocomial infections. Our study aims to isolate and identify the germs involved in community-acquired peritonitis in order to assess their susceptibility to the antibiotics available in our country.

**Methods.** This is a retrospective study of the bacteriological profile of community peritonitis in Rabat Morocco. A total of 150 adult patients with peritonitis were admitted and samples were collected intraoperatively for bacteriological examination between 1 July 2022 and 30 April 2023.

**Results.** Among the 150 patients, 101 (67.8%) were males and 49 (32.2%) were females, with a sex ratio (M/F) of 2 : 1. The mean age of the patients was 40.5 years±20.12. The distribution of bacteria was dominated by *Escherichia coli* (44%). Overall, 70% of *E. coli* isolated exhibited resistance to ampicillin, whereas no resistance to ampicillin has been reported for *Enterococcus*.

**Discussion.** In the present study, we were interested in the bacteriological profile of community peritonitis in order to adapt the antibiotic therapy to our bacterial ecology. Our findings indicate a concerning trend of increasing resistance among *E. coli* to the commonly used amoxicillin/clavulanic acid combination in our clinical setting.

**Conclusion.** Consequently, there is a need to reassess the empiric antibiotic prescribed for the management of community-acquired peritonitis.

## Data Summary

We have developed an operating database with the various variables necessary for our study. The supplementary materials have been provided in a separate Excel file. Statistical analysis used the usual description criteria of numbers and percentages.

## Introduction

Peritonitis is an acute inflammation of the peritoneum of either infectious or non-infectious origin. It is most often secondary to the perforation of a digestive tract and/or the dissemination of a septic focus within the intra-abdominal cavity. Peritonitis is considered generalized when it extends throughout the peritoneal cavity [1].

The micro-organisms involved mainly belong to the digestive flora; however, Gram-positive cocci and yeasts can be isolated in nosocomial infections [2].

Previous reports indicate a predominance of *Escherichia coli*, accounting for 65%, mostly sensitive to ceftriaxone, amoxicillin/clavulanic acid combination and imipenem [3].

Peritonitis represents a therapeutic emergency, as it can jeopardize the patient’s prognosis. Treatment involves a combination of medical and surgical interventions [4].

In Morocco, few studies have focused on the microbiological profile of the micro-organisms implicated in community peritonitis.

The main objective of this study is to isolate and identify the responsible bacteria and assess their antibiotic sensitivity profiles within our context.

## Methods

This is a retrospective study carried out in the microbiology laboratory of Military Hospital of Instruction Mohamed V, over a period of 10 months from 1 July 2022 to the end of April 2023 including 150 adult patients of both sexes aged over 15 years operated for peritonitis confirmed intraoperatively. We excluded patients under the age of 15 years, who received pre-admission antibiotic treatment and whose appendix was macroscopically healthy.

Different samples were taken, depending on the anatomical site reached, such as deep pus and peritoneal fluid. The samples received in the laboratory have benefited from a macroscopic examination (we noted their colour, clarity and viscosity). The macroscopic aspect can help to distinguish an exudate from a transude, a microscopic examination after Gram staining. The cultures were carried out on Columbia agar with 5% blood (GS), Polyvitex chocolate agar (GSC) and Sabouraud-Chloramphenicol agar (for yeast research). All these media were incubated at 37.8 °C for 18–24 h in atmospheres enriched with 5–10% CO_2_. Selective and specific media were used, such as ANC blood agar (nalidixic acid and colistin) and Schaedler agar (for anaerobic bacteria) with anaerobic incubation for 48 h. The isolated organisms were identified using conventional bacteriological methods.

The study of antibiotic sensitivity was carried out by the Muller–Hinton agar diffusion technique according to the recommendations of the Antibiotic Susceptibility Committee of the French Society of Microbiology EUCAST 2022.V.1.0 [5]. We were able to calculate the percentage of antibiotic resistance from the absolute values collected while reading the inhibition zones by measuring the diameters on the Muller–Hinton agar.

Data from the study were analysed by using the software SPSS V.20.0. Statistical analysis used the usual description criteria of numbers and percentages.

## Results

**Fig. 1.**
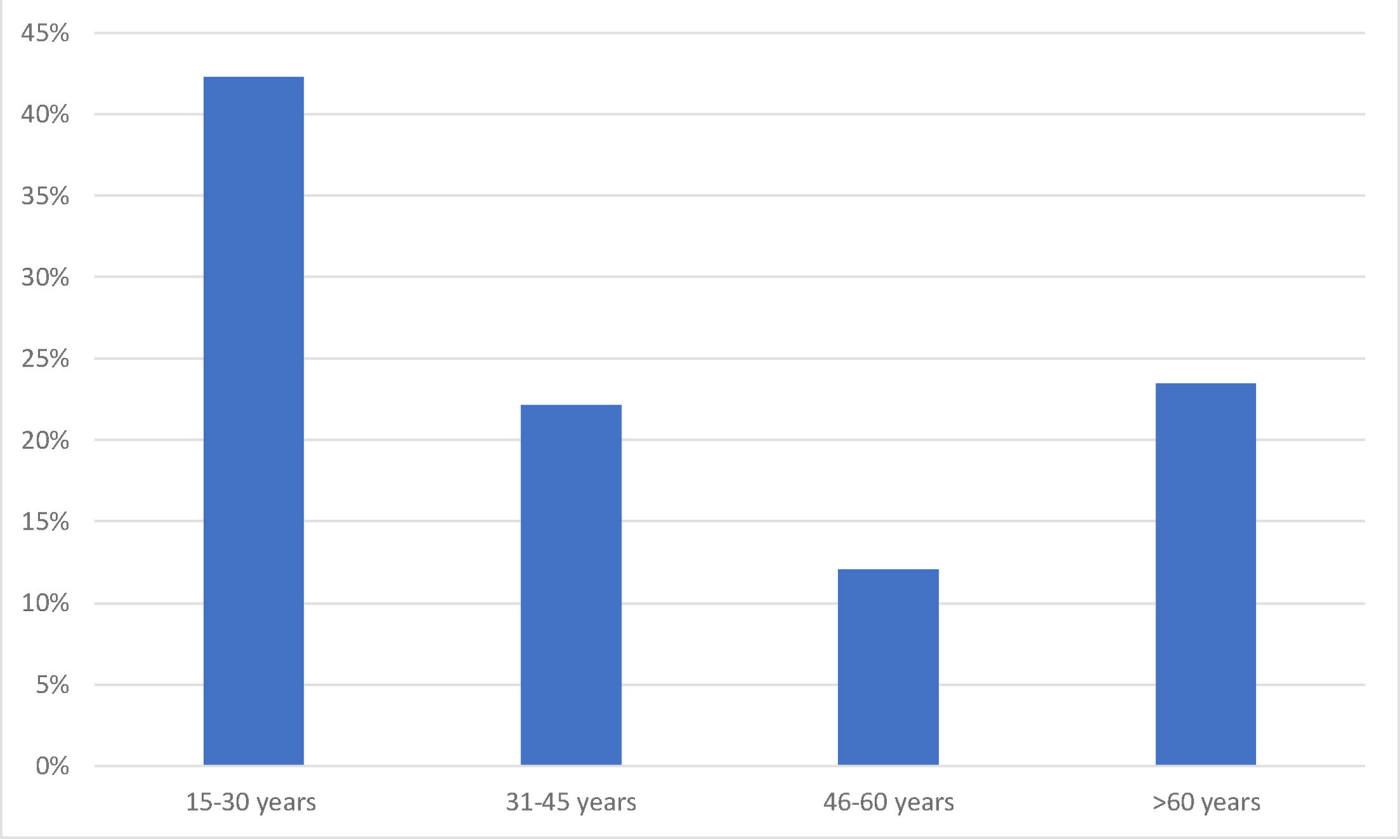
The distribution of the study population by age group.

During our study, 251 samples have been received including 150 patients, of which 162 were positive (peritonitis is related to an infection involving a bacterium), with a positivity rate of 65%. Total of 101 patients (67.8%) were males and 49 patients (32.2%) were females, and the sex ratio (M/F) was 2 : 1. The mean age of the population was 40.5 years±20.12 with extremes ranging from 15 to 94 years (Fig. 1).

The distribution of germs showed a predominance of *E. coli* with a prevalence rate of 44%, followed by *Enterococcus faecalis* (11%), *Klebsiella pneumoniae* (7%), *Proteus mirabilis* 5%, *Enterobacter cloacae* (4%) and other bacteria for the rest of the percentage.

**Fig. 2.**
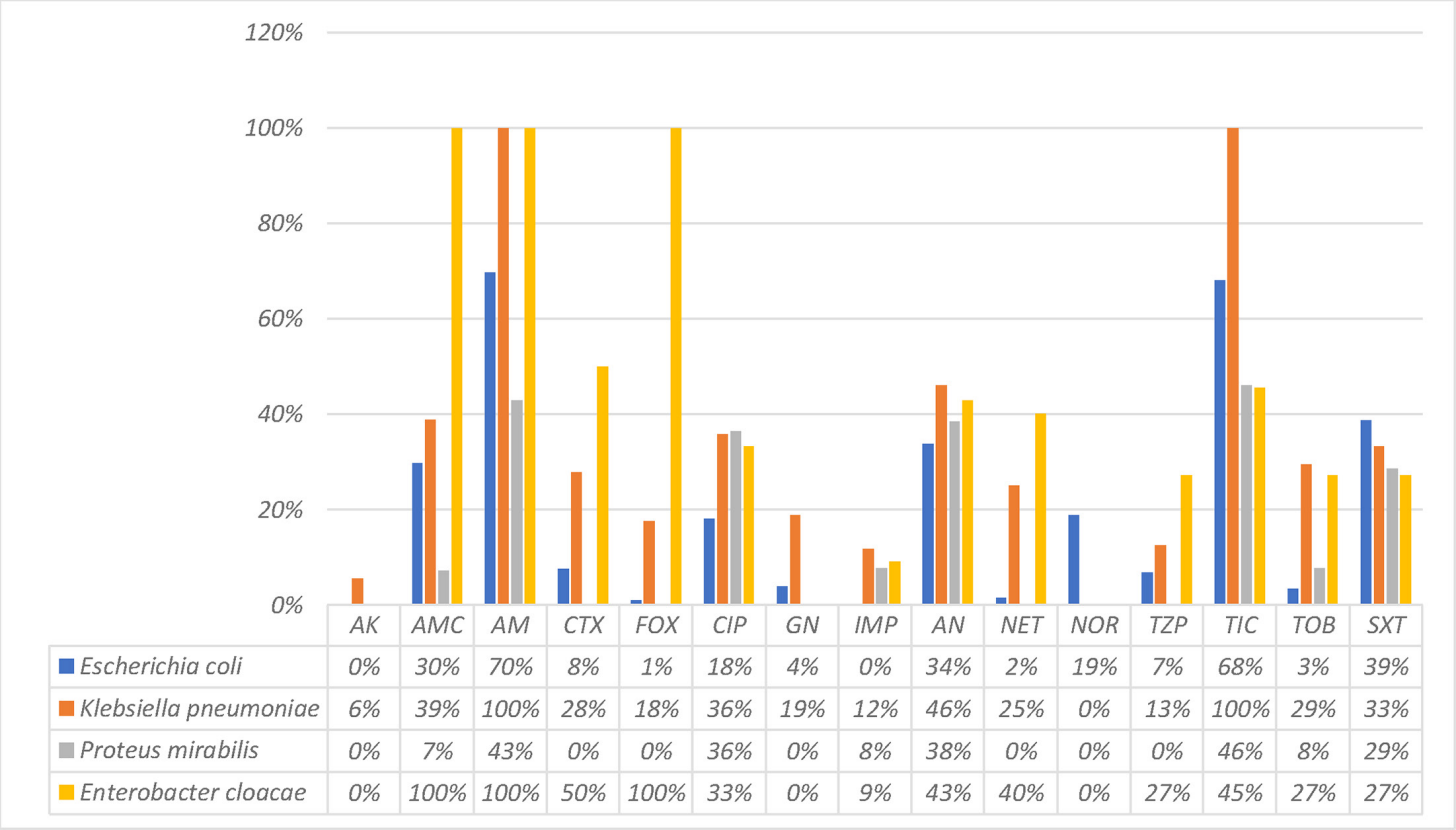
Sensitivity profile of isolated germs to antibiotics. AK: amikacin, AMC: amoxicillin+clavulanic acid, AMP: ampicillin, FEP: cefepime, CTX: cefotaxime, FOX: cefoxitin, CIP: ciprofloxacin, CN: gentamicin, IMP: imipenem, NA: nalidixic acid, NET: netilmicin, NOR: norfloxacin, TZP: piperacillin+tazobactam, TIC: ticarcillin, TM: tobramycin, SXT: trimethoprim/sulfamethoxazole.

In terms of antibiotic sensitivity, *E. coli* isolates showed a resistance rate of 70% to ampicillin, 30% to amoxicillin/clavulanic acid, 1% to cefoxitin, 18% to ciprofloxacin, 4% to gentamicin, 0% to amikacin, 30% to trimethoprim/sulfamethoxazole and 0% to imipenem (Fig. 2).

**Fig. 3.**
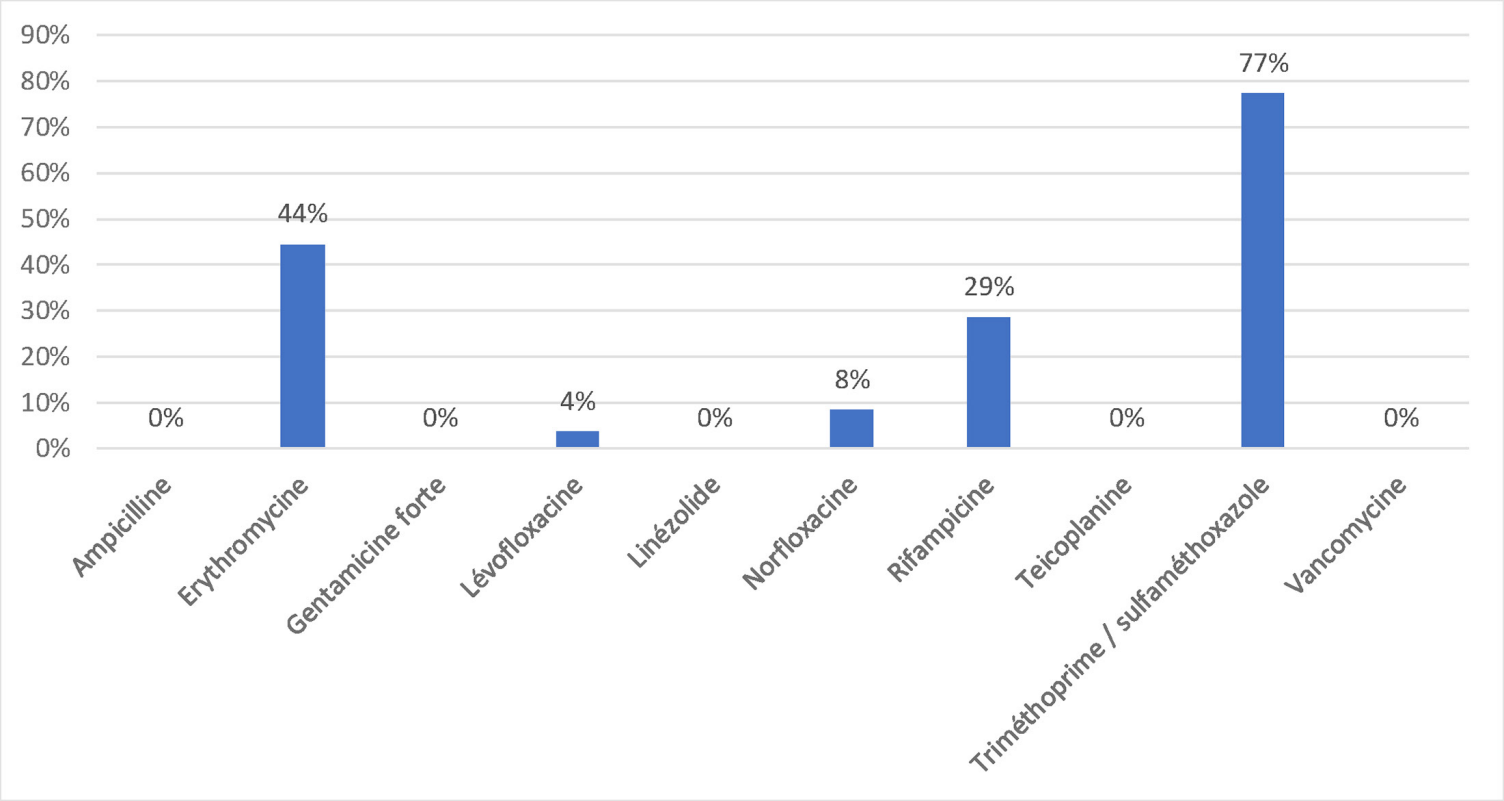
Sensitivity profile of *Enterococcus faecalis*.

As for *E. faecalis* isolates, no resistance was observed to ampicillin, gentamicin, teicoplanin or vancomycin, and it has been given its own antibiotic resistance figure according to the recommendations of the antibiotic susceptibility committee of the French Society of Microbiology EUCAST 2022.V.1.0 (Fig. 3).

## Discussion

Peritonitis is an inflammation of the double peritoneal membrane, caused in the majority of cases by bacterial infections [6]. In developing countries, bacterial peritonitis is associated with a high risk of mortality. Effective microbiological diagnosis followed by appropriate antibiotic therapy improves treatment outcomes.

The average age of our patients, 40.5 years, does not differ from that found in the literature [7].

In our study, we found a male predominance. This finding has also been reported by other authors [7,8].

The diagnosis of peritonitis is based essentially on data from the interview and clinical examination, supplemented by paraclinical examinations and confirmed at laparotomy [4].

The microbiology of peritonitis is derived from intestinal flora [9]. It often involves polymicrobial infections, but only a small number have been proven to play a pathogenic role. Enterobacteriaceae, especially *E. coli*, contribute to early mortality, while anaerobes are implicated in abscess formation [10]. These are the bacteria to be systematically considered in community-acquired peritonitis.

The most frequent microbial agents found in our study were *E. coli* (44%), *E. faecalis* (11%), *K. pneumoniae* (7%) and *Pseudomonas aeruginosa* (7%).

Our data are close to those reported in France who have found a predominance of enterobacteria, particularly *E. coli*, with prevalences of 33 and 25%, respectively. In contrast, in the USA found a predominance of *Bacteroides* sp., with a prevalence of 27%, but in less severe peritonitis. In another study conducted in the USA, *E. coli* represented 17%, while *Bacteroides* sp. accounted for 27% in peritonitis. On the other hand, *K. pneumoniae, Enterobacter* sp.*, Aerobacter* sp. and anaerobes were isolated in peptic ulcer perforation peritonitis [11,13].

Several treatment protocols have been proposed since the early 1960s [3], and numerous publications have examined the resistance profile of *E*. *coli* in peritonitis [10,14].

In our study, *E. coli* strains showed a resistance rate to amoxicillin/clavulanic acid of 30%. This rate is similar to those reported at the national level [3,12].

In an adult study conducted in 2009, 26% of *E. coli* were resistant to the combination of amoxicillin and clavulanic acid [15], revealing a 10% increase in this rate compared with a similar study conducted in 2006.

We believe that this rate of resistance in our context could be linked to the overuse of antibiotics, especially amoxicillin/clavulanic acid, often self-medicated for respiratory, digestive and urinary tract infections. This could lead to an increase in the rate of resistant strains.

New protocols for probabilistic antibiotic therapy have been proposed to treat these potentially serious infections. A triple combination of ceftriaxone, metronidazole and gentamicin is effective against *E*. *coli* and anaerobes, while ertapenem monotherapy is also effective. The use of other antimicrobials such as imipenem, cefepime, aztreonam and tigecycline must be limited to avoid the emergence of multi-resistant strains [14,16].

## Conclusion

Our study reviewed the resistance profile of *E. coli* during community-acquired peritonitis across the Rabat region of Morocco. Our results showed that this germ is becoming increasingly resistant to the amoxicillin/clavulanic acid combination, which is the antibiotic commonly used in our context. Particular attention needs to be paid to reducing the inappropriate use of antibiotics and banning self-medication. Other studies need to be carried out to monitor changes in the bacteriological profile of the germs responsible for peritonitis and to guide probabilistic antibiotic therapy.

## supplementary material

10.1099/acmi.0.000816.v5 Uncited Supplementary Material 1.

## Abbreviations

ANC: nalidixic acid and colistin
GS: Columbia agar with 5 % blood
GSC: Polyvitex chocolate agar
